# Phenotypic Characterization of Multidrug-Resistant Pseudomonas aeruginosa Isolated From Patients With Ventilator-Associated Pneumonia: Insights Into Resistance Mechanisms and Biofilm Formation

**DOI:** 10.7759/cureus.116262

**Published:** 2026-09-15

**Authors:** Sneha D Parit, Priyanka M Mane, Satish R Patil

**Affiliations:** 1 Department of Microbiology, Krishna Institute Of Medical Sciences, Krishna Vishwa Vidyapeeth (Deemed to be university), Karad, IND; 2 Department of Microbiology, Krishna Institute Of Medical Sciences, Krishna Vishwa Vidyapeeth (Deemed to be University), Karad, IND

**Keywords:** antimicrobial susceptibility, biofilm formation, extended-spectrum β-lactamase (esbl), intensive care unit (icu), metallo-β-lactamase (mbl), multidrug resistance (mdr), pseudomonas aeruginosa, ventilator-associated pneumonia (vap)

## Abstract

Background

In patients receiving mechanical ventilation in intensive care units (ICUs), ventilator-associated pneumonia (VAP) is a serious illness connected with healthcare. *Pseudomonas aeruginosa *(*P. aeruginosa*)is among the most often used pathogens linked to VAP and is increasingly characterized by multidrug resistance, β-lactamase production, and biofilm-forming ability. This study focused on assessing the antimicrobial susceptibility pattern, prevalence of multidrug resistance, extended-spectrum β-lactamase (ESBL) production, metallo-β-lactamase (MBL) production, and biofilm-forming ability among isolates of *P. aeruginosa* collected from endotracheal tube (ETT) aspirates of individuals with VAP.

Methods

A descriptive cross-sectional research study was carried out in the Microbiology Department at Krishna Charitable Hospital and Medical Research Center, Karad, India. A total of 104 non-repetitive *P. aeruginosa* isolates acquired from ETT aspirates of patients clinically suspected of VAP were included. Identification was carried out utilising conventional microbiological and biochemical methods. Antimicrobial susceptibility testing was performed using the Kirby-Bauer disk diffusion method. ESBL and the production of MBLs were detected phenotypically by combined disk diffusion methods. The Congo red agar (CRA) method and the Christensen’s tube method were used to evaluate biofilm development.

Results

Among the 104 VAP patients, 72 (69.23%) were male, and 32 (30.77%) were female. The highest proportion of cases was observed in the 21-40-year age group (45.65%). Most (49.04%) of VAP cases occurred in the medical intensive care unit (MICU), and 54.81% of cases appeared five days after starting mechanical ventilation. Antimicrobial susceptibility testing revealed complete resistance to piperacillin and imipenem (100% each), followed by piperacillin-tazobactam and meropenem (98.08% each), ciprofloxacin (97.12%), ceftazidime (95.19%), levofloxacin (94.23%), and cefepime (87.50%). Aztreonam demonstrated the highest susceptibility (83.65%). ESBL production was detected in 7 (6.73%) isolates, whereas MBL production was identified in 80 (76.92%) isolates. Biofilm formation was observed in 54 (51.92%) isolates by the Christensen’s tube method and in 23 (22.12%) isolates by the CRA method.

Conclusions

A significant occurrence of multidrug-resistant *P. aeruginosa* was found among those who had VAP. Extensive antimicrobial resistance, high MBL production, and frequent biofilm formation highlight the therapeutic obstacles linked to these infections. Continuous surveillance of antimicrobial resistance, strict infection-control measures, and judicious antibiotic use are essential to reduce the transmission of resistant strains and improve patient outcomes.

## Introduction

Critically sick patients in the intensive care unit (ICU) who are on mechanical ventilation are susceptible to ventilator-associated pneumonia (VAP), a serious infection acquired in medical settings [1]. It is defined as nosocomial pneumonia occurring at least 48 hours after endotracheal intubation and initiation of mechanical ventilation [2,3]. VAP remains among the most prevalent healthcare-associated infections worldwide and is linked to substantial Illness and fatality rates. It contributes to prolonged duration of mechanical breathing support, prolonged ICU admissions, increased healthcare costs, and an increased likelihood of fatalities in severely ill patients [1-4]. The etiological agents accountable for VAP differ greatly from those resulting in community-acquired pneumonia, with *Pseudomonas aeruginosa* (*P. aeruginosa*) and other multidrug-resistant (MDR) pathogens being among the most frequently isolated organisms [3]. There are notable regional differences in the occurrence and incidence of these high-risk pathogens, influencing local epidemiological patterns and treatment strategies [3].

*P. aeruginosa* is a non-fermenting, aerobic, Gram-negative bacillus and a key opportunistic pathogen accountable for healthcare-associated infections worldwide. In critical care settings, it is frequently implicated as a leading etiological agent of VAP [3]. The organism possesses remarkable intrinsic resistance mechanisms and environmental adaptability, enabling efficient colonization of the respiratory tract and persistence within the hospital environment. Consequently, infections due to *P. aeruginosa* are linked to unfavorable clinical results and a substantial therapeutic burden [1,3]. Patients who develop VAP due to MDR organisms, particularly *P. aeruginosa*, are often at greater risk of adverse clinical outcomes, including treatment failure and increased mortality [3].

The management of *P. aeruginosa*-associated VAP has grown more difficult due to the rising prevalence of antimicrobial resistance (AMR) in ICUs. Patients affected by MDR strains often face limited treatment options, extended hospital stays, heightened medical expenses, and higher mortality rates [3]. Of particular concern is the growing prevalence of carbapenem resistance among clinical isolates. Carbapenems are considered critical agents for the treatment of severe nosocomial infections; however, their effectiveness is increasingly threatened by the emergence of carbapenem-resistant strains [4,5]. Among the various mechanisms contributing to resistance to antimicrobials in *P. aeruginosa* is the synthesis of β-lactamase enzymes. It is among the most clinically significant. Extended-spectrum β-lactamases (ESBLs) confer resistance to a very extensive range of β-lactam antibiotics, whereas metallo-β-lactamases (MBLs) are zinc-dependent enzymes capable of hydrolyzing carbapenems and most other β-lactam agents, thereby severely restricting available therapeutic options [4,5].

The establishment of biofilms signifies another crucial virulence factor of *P. aeruginosa* [6]. The organism readily adheres to the surfaces of endotracheal tubes and other respiratory devices, producing a protective extracellular polymeric matrix that safeguards bacterial cells from the defenses of the host immune system and antimicrobial agents [6]. Biofilm-associated infections are difficult to eradicate and contribute significantly to persistent colonization, chronic infection, and therapeutic failure in mechanically ventilated patients [6]. Since ESBL production, MBL-mediated carbapenem resistance, and biofilm formation collectively enhance the persistence and dissemination of MDR *P. aeruginosa*, continuous surveillance of local resistance trends is crucial for efficient infection management and therapeutic management [5,6].

Therefore, the present study was conducted to isolate and identify *P. aeruginosa* from endotracheal aspirates of patients clinically suspected of VAP, evaluate the antimicrobial susceptibility patterns of the isolates, determine the prevalence of multidrug resistance, ESBL and MBL production, and assess biofilm-forming ability among MDR *P. aeruginosa* isolates [4-10].

## Materials and methods

Study design and setting

The present cross-sectional descriptive study was conducted in the Microbiology Department at Krishna Charitable Hospital and Medical Research Center, Karad, Maharashtra, India, from April 2024 to April 2025. Ethical approval for the study was obtained from the Institutional Ethics Committee (IEC) of Krishna Vishwa Vidyapeethd (Deemed to be University), Karad (IEC No.: KVV/IEC/05/2024).

Inclusion and exclusion criteria

All non-repetitive *P. aeruginosa* isolates recovered from endotracheal tube (ETT) aspirates of patients clinically suspected of VAP were included in the microbiological analysis. Non-*P. aeruginosa* isolates and duplicate isolates obtained from the same patient were excluded. The study focused on the microbiological characterization of *P. aeruginosa* isolates from patients clinically suspected of VAP and did not prospectively apply a standardized composite clinical, radiological, and ventilatory VAP diagnostic algorithm.

Collection and processing of specimens

Specimens were collected in a sterile manner using a sterile 22-inch, 12F suction catheter inserted through the endotracheal tube to a depth of approximately 30 cm. Gentle aspiration was performed, and the collected specimen was transferred into a sterile mucus collection tube and immediately moved to the microbiology lab for processing. From each collected sample, smears were prepared and subjected to Gram staining for preliminary microscopic examination. The morphological characteristics of the organisms were observed and recorded for further identification.

Isolation and identification of *P. aeruginosa*

Biochemical Reactions

Nutrient agar, blood agar, MacConkey agar, and cetrimide agar were used to cultivate all ETT aspirate specimens, which were then aerobically incubated at 37°C for 18-24 hours. Pure colonies obtained were subjected to Gram staining and examined for characteristic colony morphology. Presumptive identification of *P. aeruginosa* was further confirmed by standard biochemical tests, including oxidase, catalase, citrate utilization, urease hydrolysis, indole production, methyl red (MR), triple sugar iron (TSI), and motility testing [11] (Table 1).

**Table 1:** Biochemical identification tests and results of Pseudomonas aeruginosa isolates. K/K: alkaline slant and alkaline butt; no gas production; no H_2_S production [11].

Biochemical tests	Results
Gram staining	Gram-negative bacilli
Motility (hanging drop)	Motile
Catalase	Positive
Oxidase	Positive
Indole	Negative
Citrate utilization	Positive
Urease hydrolysis	Positive
Triple sugar iron	K/K, no gas/no H_2_S
Methyl red	Negative
Pigment production	Blue-green pigment (pyocyanin) positive

Antimicrobial Susceptibility Testing (AST)

AST was performed using the Kirby-Bauer disk diffusion method on sterile Mueller-Hinton agar (MHA) plates according to the standard interpretive criteria laid out by the Clinical and Laboratory Standards Institute (CLSI M100, 34th Edition) [10]. Commercial antibiotic discs were sourced from HiMedia Laboratories Pvt. Ltd., Mumbai, India. Inocula matching a 0.5 McFarland turbidity standard were prepared in tryptic soy broth or sterile normal saline and spread uniformly to form a confluent lawn.

The antipseudomonal panel tested included aztreonam (30 μg), cefepime (30 μg), ceftazidime (30 μg), ciprofloxacin (30 μg), imipenem (10 μg), levofloxacin (5 μg), meropenem (10 μg), piperacillin (100 μg), and piperacillin/tazobactam (100/10 μg). The diameter of inhibitory zones was measured after 24 hours of aerobic incubation at 37°C and categorized as susceptible, intermediate, or resistant. The strain used for quality control in evaluating antimicrobial susceptibility testing was *P. aeruginosa* ATCC 27853 [7-10].

Multidrug Resistance (MDR) Categorization

Relying on the criteria mentioned by Magiorakos et al., isolates exhibiting acquired non-susceptibility to one or more antimicrobial substances that fall under three or more antibiotic groups were categorized as multidrug-resistant (MDR) [9].

Phenotypic screening

Extended-Spectrum Beta-Lactamase (ESBL) Detection

Extended-spectrum β-lactamases (ESBLs) are enzymes that hydrolyze extended-spectrum β-lactam antibiotics and are inhibited by β-lactamase inhibitory agents, including clavulanic acid. Ceftazidime discs (30 μg) used alone, along with clavulanic acid (ceftazidime/clavulanic acid, 30/10 μg), were applied onto Mueller-Hinton agar plates inoculated with the test isolate. An inhibition zone difference of at least 5 mm around the combination disc compared to the ceftazidime disc alone indicated a positive result for ESBL production. ESBL production was phenotypically identified using the combined disk diffusion method according to the protocol described by Wadekar et al. [4]. Representative phenotypic results demonstrating ESBL non-producing and ESBL-producing *P. aeruginosa* isolates are shown in Figure 1A and Figure 1B, respectively.

**Figure 1:**
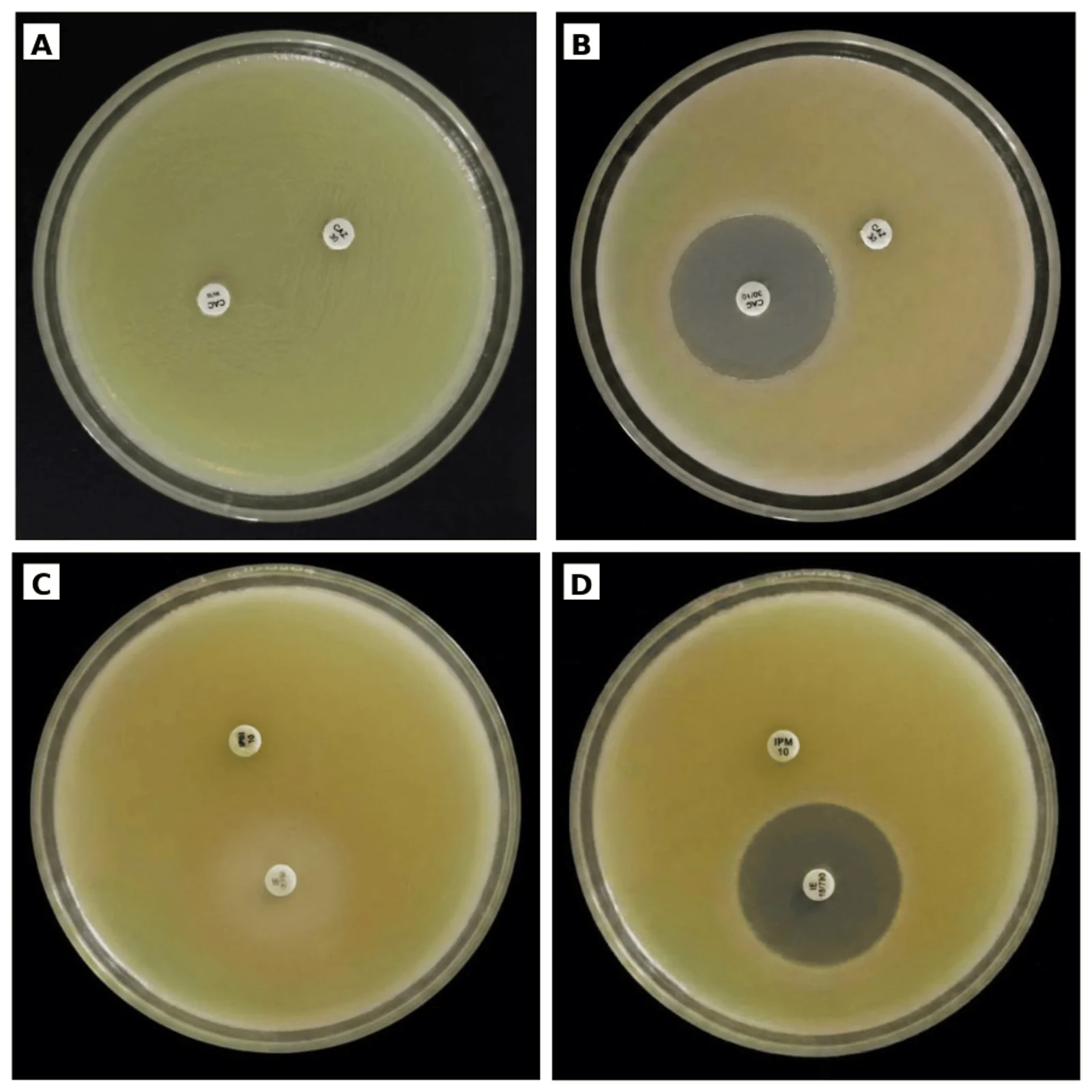
Phenotypic detection of ESBL and MBL production in Pseudomonas aeruginosa. (A) ESBL non-producing isolate; (B) ESBL-producing isolate; (C) MBL non-producing isolate; (D) MBL-producing isolate. ESBL: extended-spectrum beta-lactamase; MBL: metallo-beta-lactamase.

Metallo-Beta-Lactamase (MBL) Detection

Metallo-β-lactamases (MBLs) are zinc-dependent enzymes capable of hydrolyzing carbapenems and other β-lactam antibiotics. A 10 μg imipenem disk was placed edge-to-edge at 20 mm from a combined imipenem-EDTA disk on a Mueller-Hinton agar plate inoculated with the test isolate. Following an overnight incubation period at 37°C, the inhibitory zone diameter increased by over 7 mm around the imipenem-EDTA disk, in comparison to the imipenem disk alone, which was indicative of MBL production [7]. Representative phenotypic results demonstrating MBL non-producing and MBL-producing *P. aeruginosa* isolates are shown in Figure 1C and Figure 1D, respectively.

Phenotypic Characterization of Biofilm Production

Tube method (TM): The tube method is a qualitative screening technique employed to identify biofilm development depending on the adherence of bacterial cells to the inner surface of test tubes. Test isolates were inoculated into 10 mL of trypticase soy broth supplemented with 1% glucose using a sterile loop and incubated at 37°C for 24 hours. After incubation, the culture supernatant was removed, and the tubes were carefully washed with phosphate-buffered saline (pH 7.3). Adherent cellular layers were stained using 0.1% crystal violet solution, followed by inversion drying. The Positive establishment of biofilm was characterized by the existence of a visible, dense, stained film lining the bottom and side walls of the tube, distinguishing it from a faint line at the air-liquid interface. The qualitative tube method was carried out as described by Rewatkar & Wadher [8,9]. Representative results demonstrating biofilm-producing and biofilm non-producing *P. aeruginosa* isolates by the tube method are shown in Figure 2A and Figure 2B, respectively.

**Figure 2:**
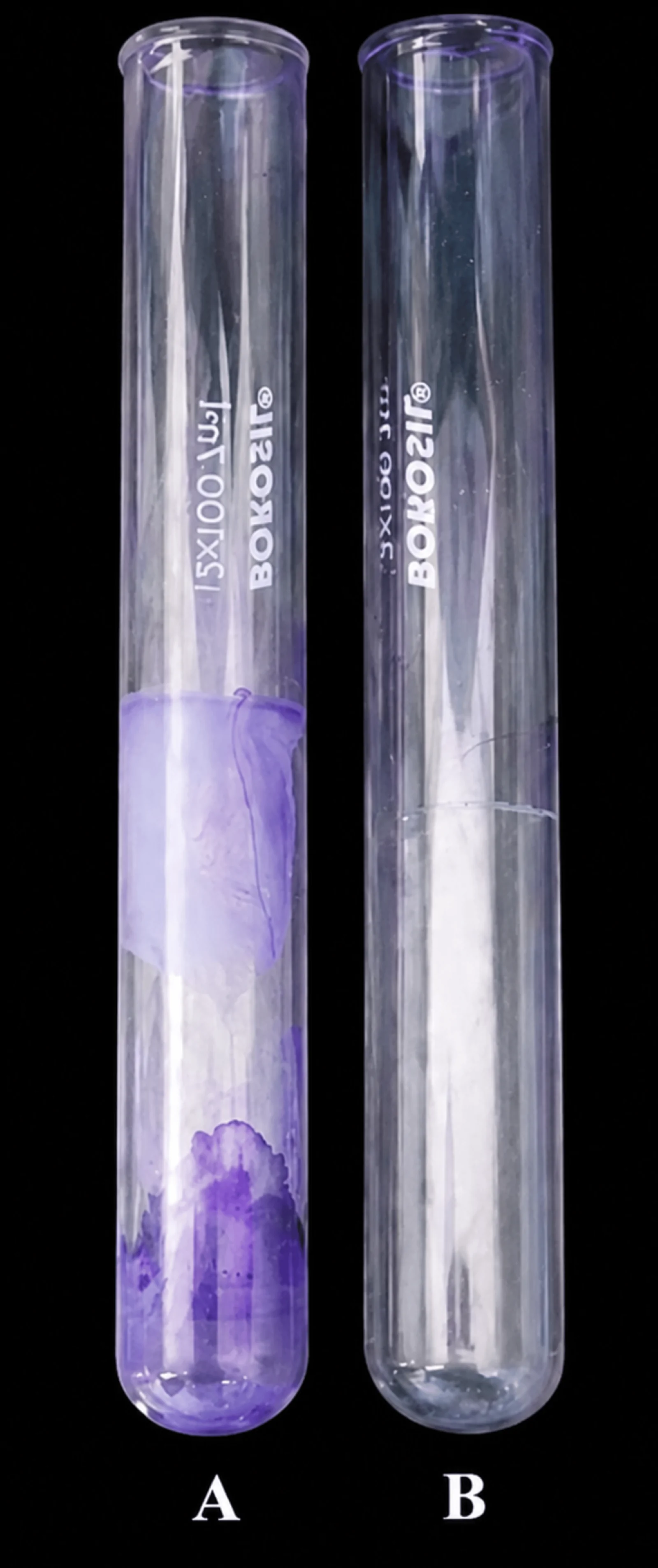
Biofilm production tube method. (A) Biofilm producing; (B) Biofilm non-producing.

Congo red agar (CRA) method: The CRA method is a qualitative technique used to detect biofilm. Production was determined by characteristic colony morphology on a specialized agar medium. The isolates were subjected to inoculation onto CRA plates maintained under aerobic conditions at 37°C for 24-48 hours. Biofilm-producing isolates were characterized by the formation of dry, crystalline black colonies, whereas non-biofilm-producing isolates produced smooth red or pink colonies. Biofilm-forming ability was evaluated by the CRA method as described by Rewatkar & Wadher [8]. Representative results demonstrating biofilm non-producing and biofilm-producing *P. aeruginosa* isolates by the CRA method are shown in Figure 3A and Figure 3B, respectively.

**Figure 3:**
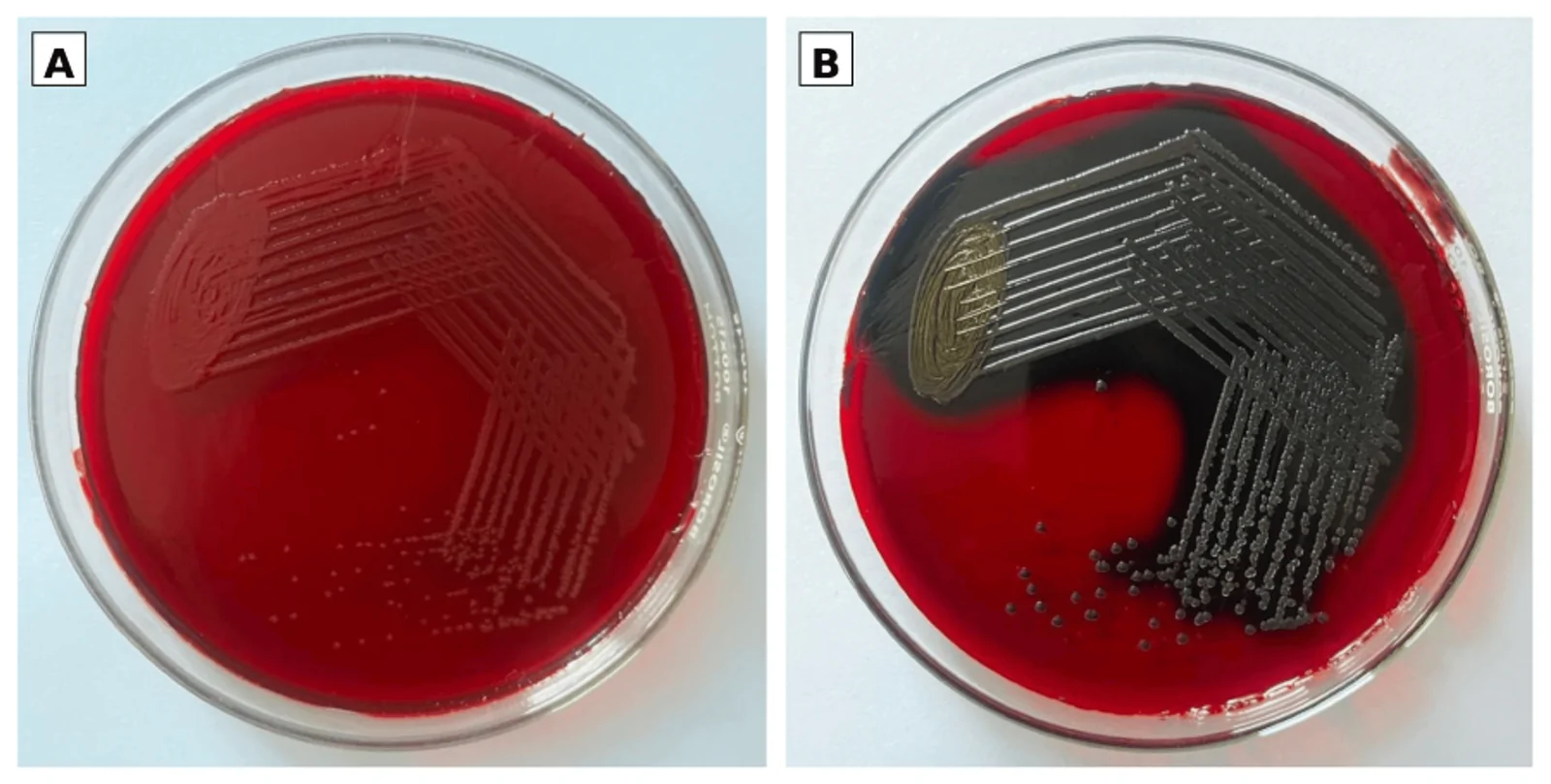
Biofilm production by the CRA plate method in Pseudomonas aeruginosa. (A) Biofilm non-producing isolate; (B) Biofilm-producing isolate. CRA: Congo red agar.

Statistical analysis

This was a descriptive cross-sectional study; only descriptive statistics (frequency and percentage) were subjected to statistical analysis, and no inferential statistical tests were applied.

## Results

A total of 104 ETT aspirate specimens obtained from patients with VAP were included in the present investigation.

Among the 104 patients with VAP, 72 (69.23%) were male, and 32 (30.77%) were female. The highest proportion of patients belonged to the 21-40-year age group, accounting for 47 cases (45.2%), followed by the 41-60-year age group with 33 cases (31.73%). Patients aged 61-80 years constituted 22 cases (21.15%), while only one case (0.96%) each was recorded in the 1-20 years and 81-100 years age groups. These findings indicate that VAP was more frequently noted among younger and middle-aged adults, with a predominance of male patients (Table 2).

**Table 2:** Age- and gender-wise distribution in VAP patients. VAP: ventilator-associated pneumonia; n: number; %: percentage.

Age group (years)	Gender		Total
	Male (n-72) (69.23%)	Female (n-32) (30.77%)	n (%)
1 to 20	0	1 (0.96)	1 (0.96)
21 to 40	35 (33.65)	12 (11.54)	47 (45.2)
41 to 60	23 (22.12)	10 (9.62)	33 (31.73)
61 to 80	13 (12.50)	9 (8.65)	22 (21.15)
81 to 100	1 (0.96)	0	1 (0.96)
Total	104 (72+32)		104 (100)

Among the 104 patients with VAP, the highest frequency of cases was found in the medical intensive care unit (MICU), accounting for 51 cases (49.04%), followed by the surgical intensive care unit (SICU) with 24 cases (23.08%). Patients in the general ICU constituted 22 cases (21.15%), while the emergency intensive care unit (EICU) recorded 6 cases (5.77%). The lowest frequency occurred least frequently in the pediatric intensive care unit (PICU), with just one reported case (0.96%) observed among the total study population. These findings indicate that VAP was common among individuals admitted to the medical and surgical intensive care units (Table 3).

**Table 3:** Distribution of ventilator-associated pneumonia (VAP) patients across intensive care units. n: number of individuals; %: percentage; PICU: pediatric intensive care unit; EICU: emergency intensive care unit; SICU: surgical intensive care unit; MICU: medical intensive care unit; ICU: intensive care unit.

Department	n	%
PICU	1	0.96
EICU	6	5.77
SICU	24	23.08
MICU	51	49.04
ICU	22	21.15
Total	104	100

Among the 104 patients with VAP, a large proportion of cases developed within ≤5 days of mechanical ventilation, accounting for 57 cases (54.81%), followed by patients ventilated for 6-10 days with 35 cases (33.65%). A smaller proportion of cases were recorded among patients who were ventilated for a period of >10 days, accounting for 12 cases (11.54%). These findings indicate that the majority of VAP cases occurred early during the course of mechanical ventilation (Figure 4).

**Figure 4:**
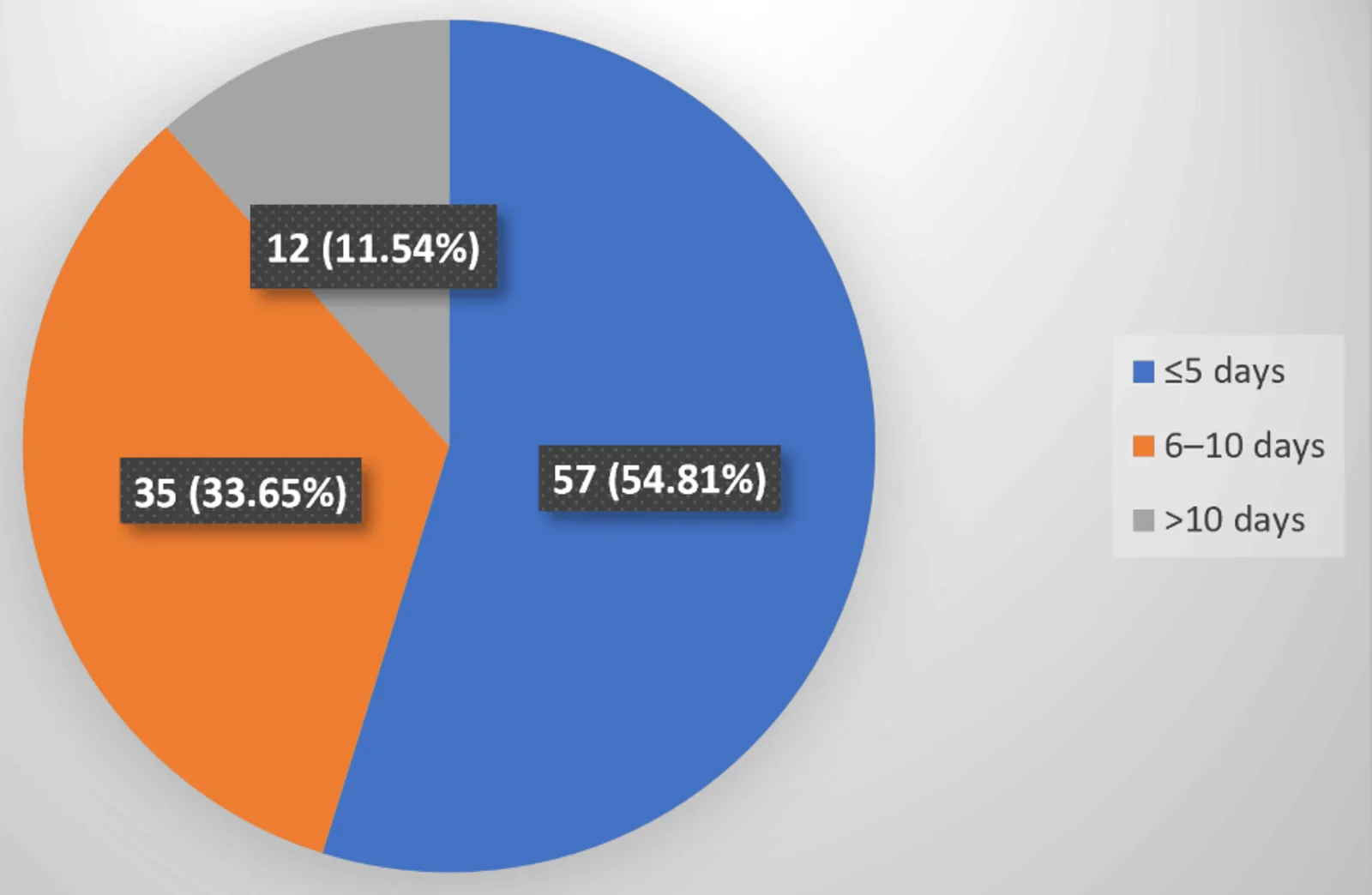
Distribution of patients with VAP according to duration of mechanical ventilation. VAP: ventilator-associated pneumonia.

Among the 104 patient-derived *P. aeruginosa* isolates from cases of VAP, the greatest resistance was noted against piperacillin and imipenem, with all isolates exhibiting resistance (100%, 104 cases each), followed by piperacillin-tazobactam (98.08%, 102 cases), meropenem (98.08%, 102 cases), ciprofloxacin (97.12%, 101 cases), ceftazidime (95.19%, 99 cases), levofloxacin (94.23%, 98 cases), and cefepime (87.50%, 91 cases). In contrast, aztreonam showed comparatively higher sensitivity, with 87 sensitive isolates (83.65%) and 17 resistant isolates (16.35%). These findings indicate an extensive multidrug resistance profile among *P. aeruginosa* isolates in VAP patients (Figure 5).

**Figure 5:**
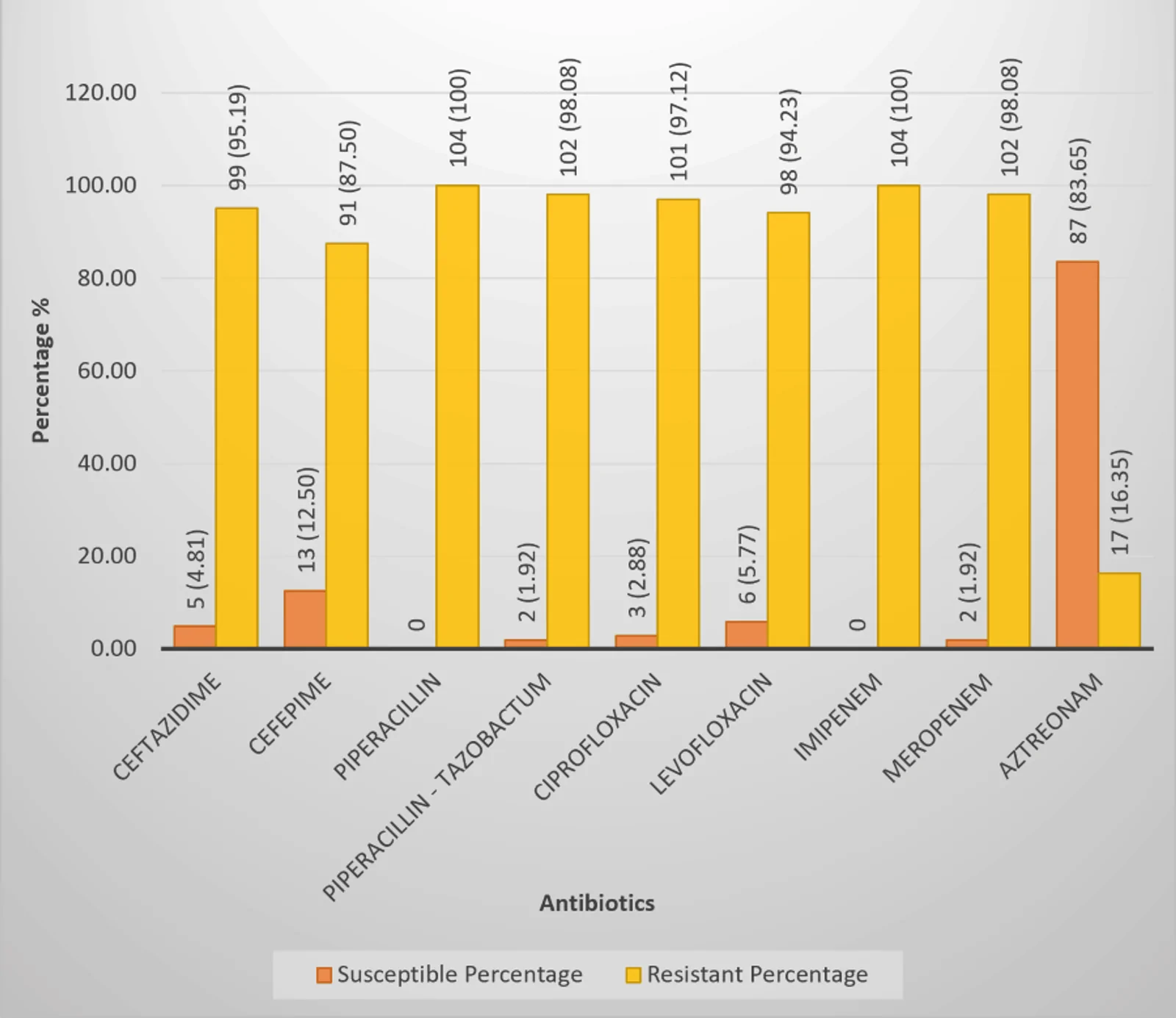
Antibiotic susceptibility profile of Pseudomonas aeruginosa isolates in VAP. VAP: ventilator-associated pneumonia.

Out of the 104 clinical *P. aeruginosa* isolates obtained from individuals with ventilator-associated pneumonia (VAP), ESBL production was observed in 7 isolates (6.73%), while the majority of the tested isolates were identified as ESBL non-producing, accounting for 92 isolates (88.46%). ESBL testing could not be performed for 5 isolates (4.81%) because they were sensitive to ceftazidime. These findings indicate a low prevalence of ESBL-producing *P. aeruginosa* among the study isolates (Figure 6).

**Figure 6:**
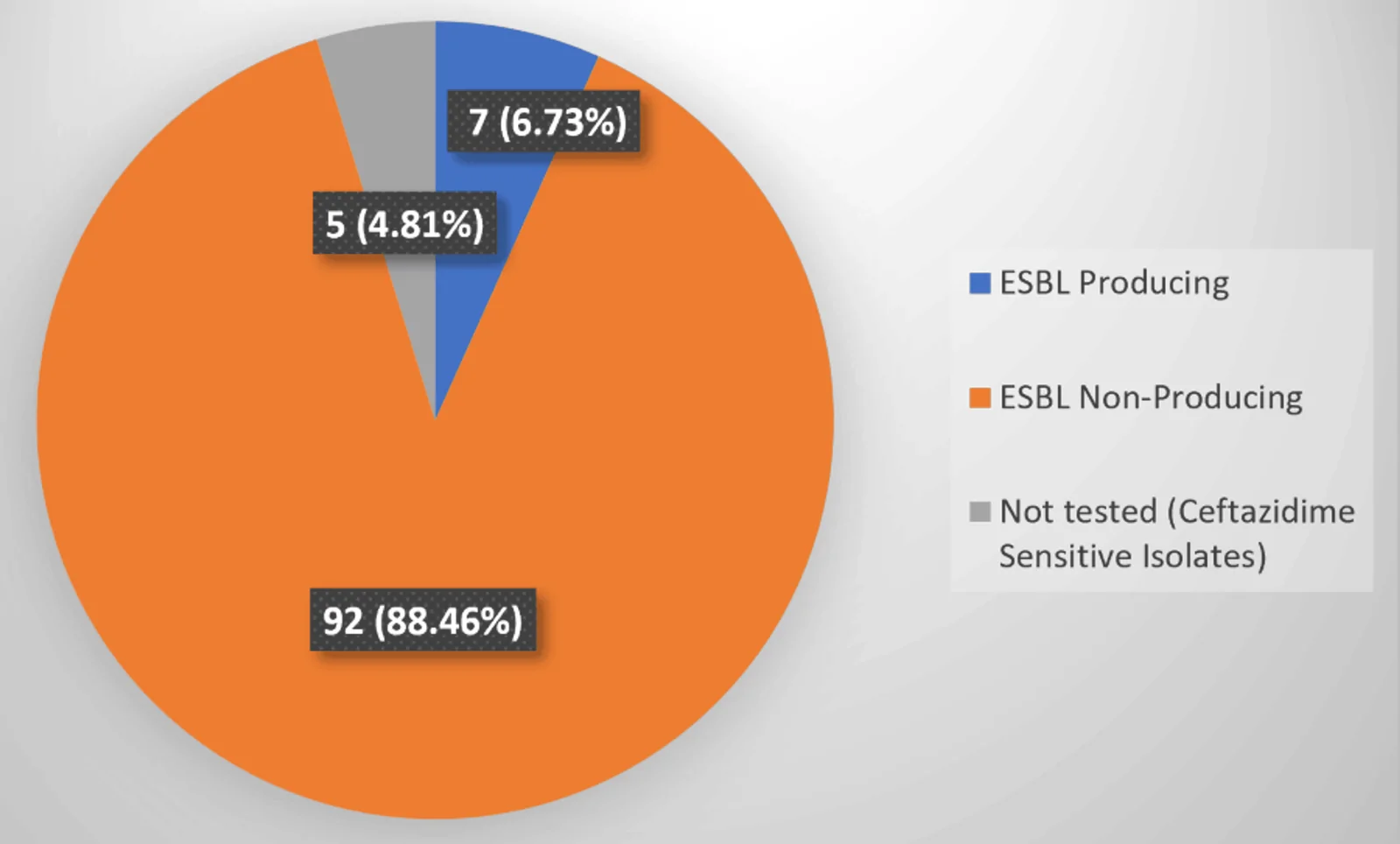
Detection of ESBL production in Pseudomonas aeruginosa isolates in VAP. ESBL: extended-spectrum beta-lactamase; VAP: ventilator-associated pneumonia.

From the 104 *P. aeruginosa* isolates derived from patients with ventilator-associated pneumonia (VAP), MBL production was identified in 80 isolates (76.92%), while 24 isolates (23.08%) were identified as MBL non-producing. These findings indicate that MBL-producing *P. aeruginosa* isolates were highly prevalent among the study isolates (Figure 7).

**Figure 7:**
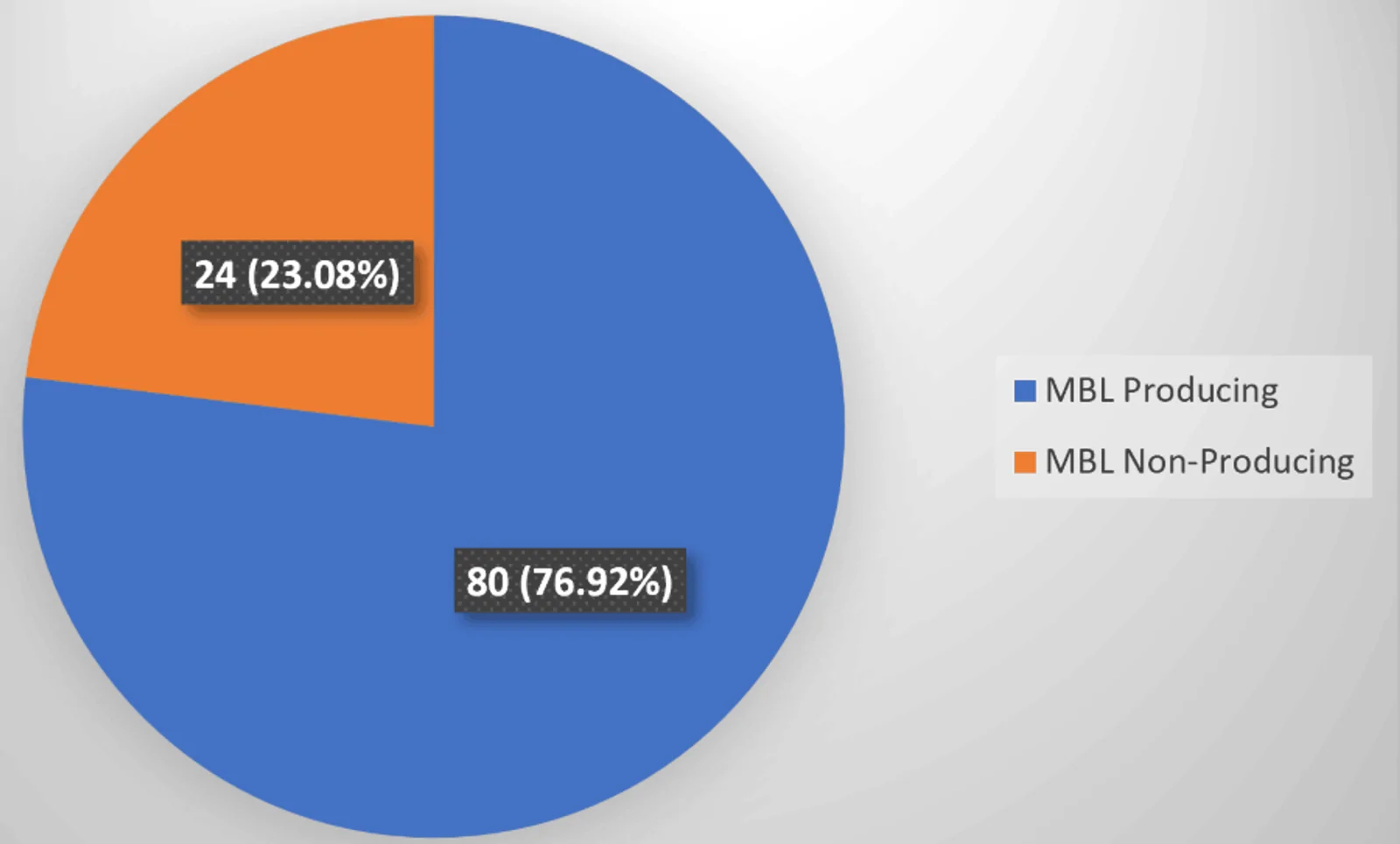
Detection of MBL production in Pseudomonas aeruginosa isolates in VAP. MBL: metallo-beta-lactamase; VAP: ventilator-associated pneumonia.

Overall, 104 isolates of *P. aeruginosa* derived from patients with ventilator-associated pneumonia (VAP) were investigated for their potential to form biofilms by the Christensen’s method. Biofilm formation was exhibited by 54 isolates (51.92%), while 50 isolates (48.08%) were determined to be non-biofilm producers. These findings indicate that more than half of the isolates had the capability to create biofilms (Figure 8).

**Figure 8:**
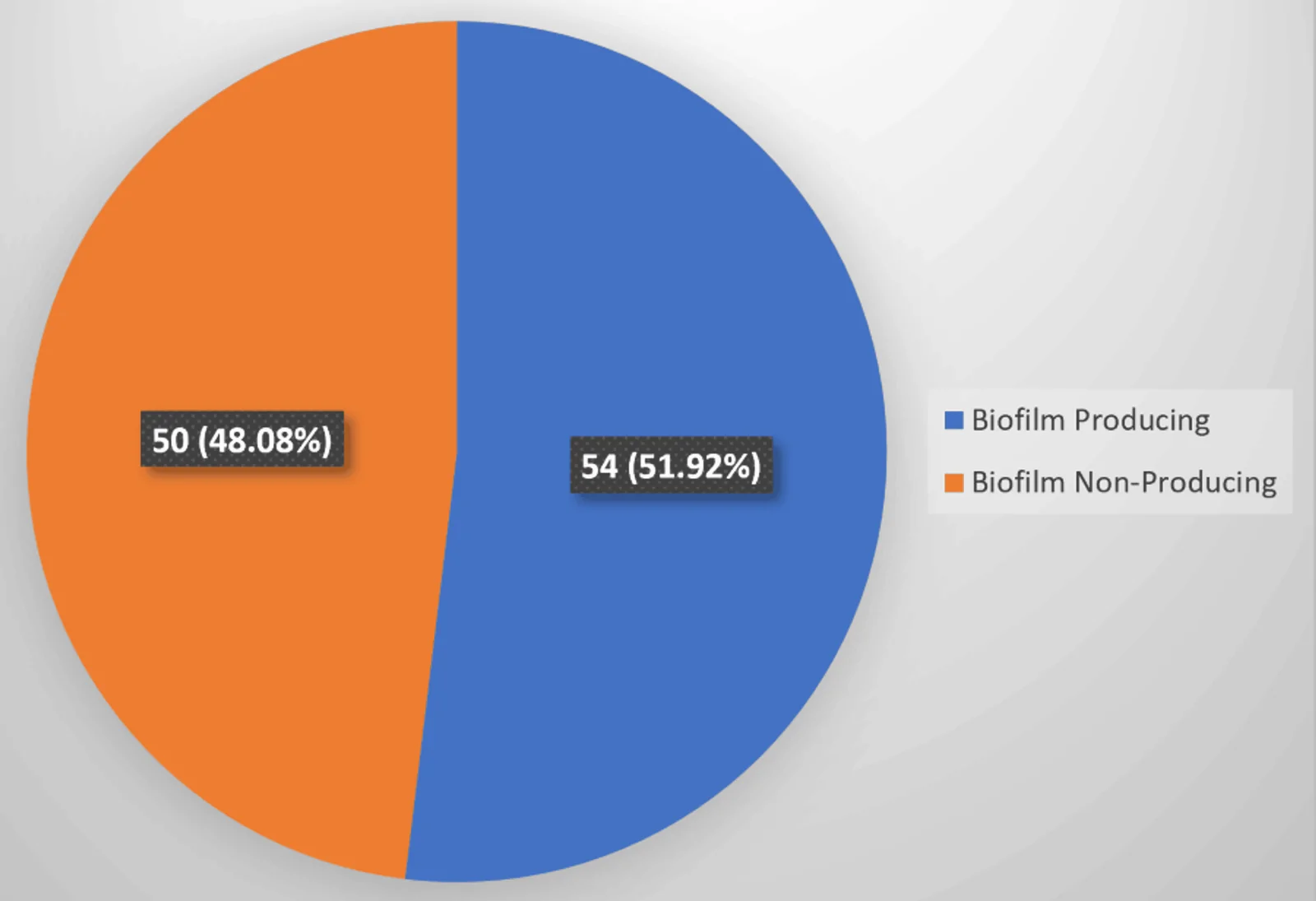
Biofilm production in Pseudomonas aeruginosa by Christensen’s tube method. %: percentage.

A total of 104 patient-derived *P. aeruginosa* isolates with ventilator-associated pneumonia (VAP) were examined using the CRA method for their capacity to produce biofilms. Biofilm-forming ability was detected in 23 isolates (22.12%), whereas 81 isolates (77.88%) were recognized as biofilm non-producers. The study findings indicate that over half of the *P. aeruginosa* isolates failed to demonstrate biofilm-forming ability by the plate method (Figure 9).

**Figure 9:**
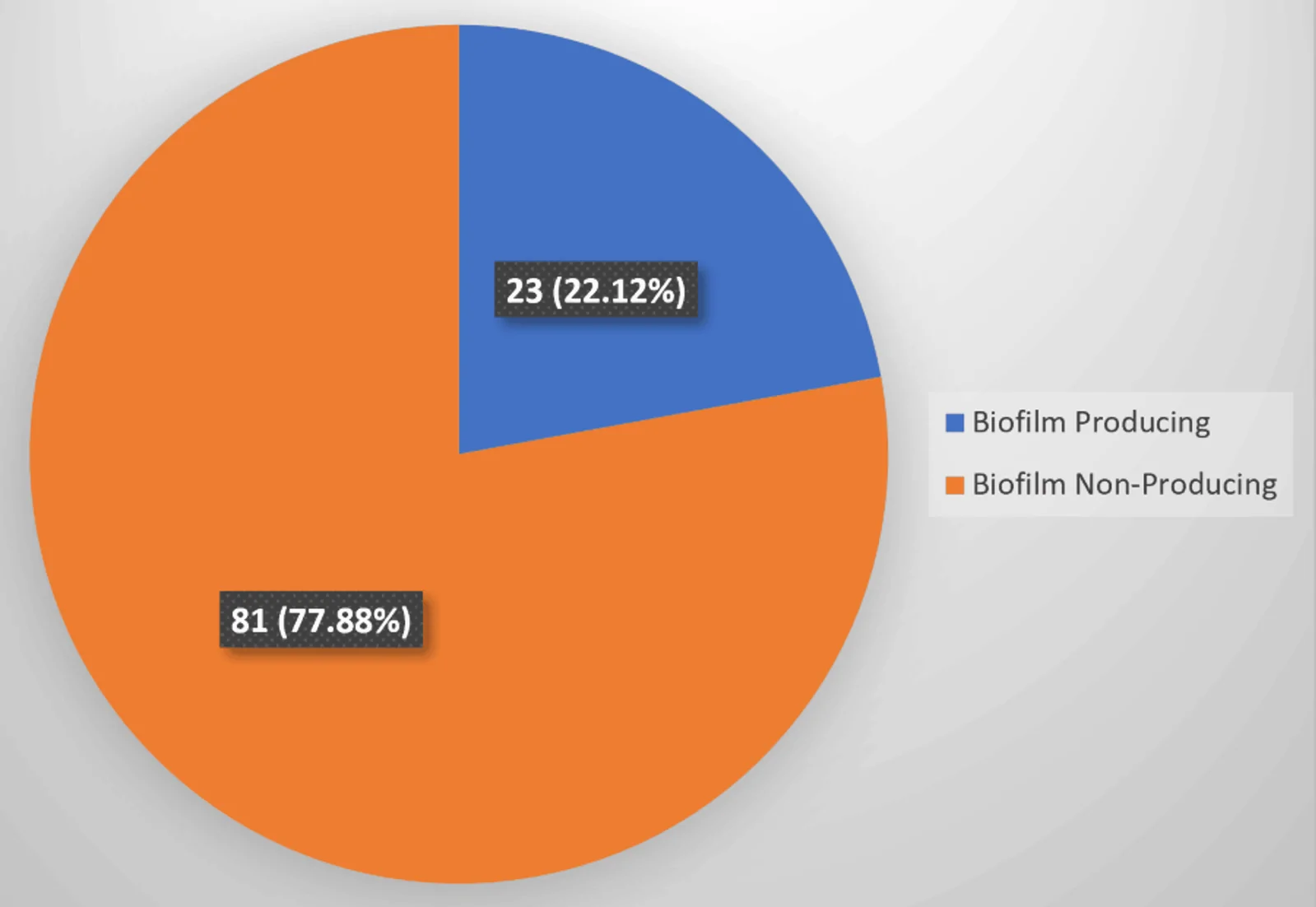
Biofilm production in Pseudomonas aeruginosa by CRA method. CRA: Congo red agar.

## Discussion

VAP continues to be frequently reported as one of the foremost healthcare-associated infections in ICUs and is linked to higher morbidity, mortality, extended duration of hospitalization, and increased healthcare costs [12,13]. Patients receiving ventilatory support are particularly vulnerable to VAP because endotracheal intubation compromises normal host defense mechanisms, facilitates bacterial colonization of the lower respiratory tract, and promotes the progression of respiratory infections arising from opportunistic pathogens such as *P. aeruginosa* [12,13]. The current research was undertaken to assess the demographic characteristics, antimicrobial resistance pattern, ESBL and MBL production, and biofilm-forming ability of multidrug-resistant *P. aeruginosa* detected in respiratory samples collected via endotracheal aspiration among patients with VAP.

Among the patients clinically suspected of VAP included in the present study, a higher proportion were male (69.23%) than female (30.77%). The highest number of cases was recorded in the 21-40 years age group (45.65%), followed by the 41-60 years age group (32.12%). Consistent results have also been observed by Joseph et al. and Gadani et al., who documented a predominance of male patients among VAP cases [14,15]. The higher frequency among males may be related to greater exposure to risk factors such as trauma, smoking, chronic illnesses, and ICU admissions [15]. The findings of the present study suggest that the predominance of young and middle-aged adults may be associated with the demographic characteristics and admission practices of the study center.

Analysis of ICU-wise distribution highlighted that the majority of VAP cases occurred in the MICU (49.04%), followed by the SICU (23.08%). The study findings correspond with earlier studies by Othman & Abdelazim, who demonstrated a higher burden of VAP in patients in critical condition requiring admission to medical and surgical intensive care units [16]. The rise in incidence in these units may be attributed to prolonged mechanical ventilation, greater disease severity, and frequent exposure to invasive procedures and broad-spectrum antibiotics [13,16].

The period of ventilator support is a well-recognized risk factor for VAP development. The results of this investigation showed that more than half of the VAP cases (54.81%) developed within the first five days of mechanical ventilation, while 33.65% occurred between 6 and 10 days. These findings are consistent with reports by Kalanuria et al. and Papazian et al., who emphasized that microbial colonization of endotracheal tubes begins soon after intubation and can rapidly progress to lower respiratory tract infection [12,13]. Early development of VAP in the present investigation may be associated with aspiration of contaminated secretions and early bacterial colonization of the airway [12,13].

The antimicrobial susceptibility distribution demonstrated an alarming level of the prevalence of resistance among the *P. aeruginosa* isolates. Complete resistance was noted against piperacillin and imipenem, while extremely elevated resistance characteristics were analysed for piperacillin-tazobactam, meropenem, ciprofloxacin, ceftazidime, levofloxacin, and cefepime. The highest susceptibility rate was noted for aztreonam among the antimicrobial agents tested. Comparable resistance patterns have been documented by Gupta et al. and Javiya et al., who documented increasing antimicrobial resistance in clinical isolates of *P. aeruginosa* [17,18]. The high resistance noted in the current research might be ascribed to extensive antibiotic exposure, selective pressure in ICU environments, and the intrinsic ability of *P. aeruginosa* to acquire various resistance mechanisms, such as efflux pumps, porin loss, enzymatic inactivation, and target-site modifications [19].

ESBL production was detected in only 6.73% of isolates in this study. This prevalence shows a reduced rate relative to findings from other geographical regions but is comparable to findings reported by Aggarwal et al., who observed a lower rate of ESBL-producing *P. aeruginosa *[20]. The comparatively reduced prevalence of ESBL production suggests that β-lactam resistance among the study isolates may be driven predominantly by alternative resistance mechanisms rather than ESBL production alone [19].

A major finding of this study was the high prevalence of MBL-producing isolates, which accounted for 76.92% of the *P. aeruginosa* isolates. Consistent findings have been observed by Manoharan et al. and Kali et al., who demonstrated a substantial burden of MBL-producing *P. aeruginosa* in intensive care settings [21,22]. The high frequency of MBL production noted in the present study likely contributed to the extensive resistance to carbapenems, particularly imipenem and meropenem. Since MBL enzymes hydrolyze a broad range of β-lactam antibiotics, their increasing prevalence poses a serious therapeutic challenge and significantly limits available treatment options [21,22].

Biofilm production evaluated using the Christensen’s method revealed that 51.92% of the isolates demonstrated biofilm production, whereas 48.08% were non-producers. The findings underscore that biofilm formation is a common characteristic among VAP-associated *P. aeruginosa* isolates. Consistent observations have been described by Rewatkar & Wadher and Tuon et al., who highlighted the ability of *P. aeruginosa* to form biofilms on medical devices and respiratory tract surfaces [8,23]. Biofilm development improves bacterial endurance by shielding microorganisms from immune reactions and antimicrobial agents, thereby contributing to persistent infections [23].

Using the CRA method, 22.12% of isolates were identified as biofilm producers, whereas 77.88% were classified as non-producers. The lower detection rate observed with the CRA method compared with the tube method may be attributed to differences in methodological sensitivity and interpretation criteria. Similar observations regarding differences between phenotypic biofilm detection methods were reported by Rewatkar & Wadher [8]. The findings suggest that the tube method may be more sensitive for detecting biofilm development among clinical isolates of *P. aeruginosa*.

An important observation obtained in the present investigation was that biofilm-producing isolates exhibited higher resistance to a greater proportion of the antimicrobial agents in comparison to non-biofilm producers. These findings are consistent with previous reports by Ciofu & Tolker-Nielsen, who demonstrated that biofilm-associated bacteria exhibit enhanced tolerance to antimicrobial agents because of restricted antibiotic penetration, altered metabolic activity, and the existence of persister cells in the biofilm matrix [24]. The coexistence of biofilm production and multidrug resistance therefore represents a major challenge in the management of *P. aeruginosa*-associated VAP [24].

Overall, the present study demonstrates a substantial burden of multidrug-resistant, MBL-producing, and biofilm-forming *P. aeruginosa *in patients presenting with VAP. The coexistence of antimicrobial resistance and biofilm-forming ability presents a considerable challenge in the management and control of these infections. Continuous surveillance of resistance patterns, routine screening for resistance mechanisms, implementation of strict infection-control measures, and rational antibiotic use are essential to mitigate the transmission of *P. aeruginosa* isolates demonstrating reduced susceptibility in intensive care units [25].

This study has several limitations. First, it was conducted at a single tertiary care center, which may limit the generalizability of the findings. Second, a standardized composite clinical, radiological, and ventilatory definition of VAP was not prospectively applied. Third, quantitative colony counts and detailed comorbidity data were not systematically recorded. Fourth, minimum inhibitory concentration (MIC) determination and molecular characterization of resistance and biofilm-associated genes were not performed. Finally, biofilm formation was assessed using qualitative phenotypic methods rather than a quantitative microtiter plate assay. Future prospective multicentric studies incorporating standardized VAP diagnostic criteria, quantitative microbiological assessment, detailed clinical risk factors, MIC determination, molecular characterization, and quantitative biofilm assessment are warranted.

## Conclusions

The present study demonstrated a high prevalence of multidrug-resistant *P. aeruginosa* among isolates recovered from endotracheal aspirates of patients clinically suspected of ventilator-associated pneumonia (VAP). Most isolates exhibited extensive resistance to frequently utilized antipseudomonal antibiotics, particularly piperacillin, imipenem, meropenem, ciprofloxacin, and levofloxacin, indicating limited therapeutic options. Metallo-β-lactamase (MBL) production was detected in a substantial proportion of isolates, whereas extended-spectrum β-lactamase production was comparatively low. Biofilm formation was frequently observed, especially when assessed by the tube method, suggesting that biofilm-associated persistence may contribute to the chronicity of infection and increased antimicrobial resistance.

The coexistence of multidrug resistance, MBL production, and biofilm-forming ability among *P. aeruginosa* isolates poses a significant challenge in the management of VAP. These findings underscore the importance of continuous surveillance of antimicrobial resistance, early detection of resistance mechanisms, strict infection-control measures, and judicious antibiotic use to restrict the dissemination of resistant strains and improve patient outcomes.
